# Synthesis and Integration of an Fe(II) Coordination Compound into Green Resin Matrices for Multifunctional Dielectric, Piezoelectric, Energy Harvesting, and Storage Applications

**DOI:** 10.3390/polym17182509

**Published:** 2025-09-17

**Authors:** Anastasios C. Patsidis, Ioanna Th. Papageorgiou, Zoi G. Lada

**Affiliations:** 1 Department of Materials, School of Natural Sciences, University of Patras, 26504 Patras, Greece; 2 Department of Chemistry, School of Natural Sciences, University of Patras, 26504 Patras, Greece; papageorgiou.ioanna@ac.upatras.gr

**Keywords:** green epoxy, iron coordination compound, synthesis, thermomechanical properties, dielectric behavior, energy storing/retrieving efficiency, piezoelectric response, sustainable materials

## Abstract

Polymer-based hybrid composites have emerged as promising platforms for multifunctional energy applications, combining structural versatility with tunable dielectric behavior. In this study, synthesized [Fe(bpy)_3_]SO_4_; (tris(2,2′-bipyridine)iron(II) sulfate) coordination compound was incorporated into a green epoxy resin matrix to fabricate nanocomposites aimed at enhancing dielectric permittivity (*ε′*), piezoelectric coefficient (*d*_33_, pC/N), energy-storage efficiency (*n_rel_*, %), and mechanical strength (*σ*, MPa). The integration of the Fe(II) complex via Scanning Electron Microscopy (SEM) confirmed a homogeneous dispersion within the matrix. Broadband Dielectric Spectroscopy (BDS) revealed the presence of three relaxation processes in the spectra of the tested systems, demonstrating enhanced dielectric permittivity with increasing Fe(II) content. Under progressively shorter relaxation times (*τ*, s), key processes such as interfacial polarization, the polymer matrix’s transition from a glassy to a rubbery state, and the dynamic reorganization of polar side groups along the polymer backbone are activated. The ability to store and retrieve electric energy was confirmed by varying filler content under direct current (*dc*) conditions. The nanocomposite with 10 phr (mass parts/100 mass parts of resin) filler achieved a piezoelectric coefficient of *d*_33_ = 5.1 pC/N, an energy-storage efficiency of *n_rel_* = 44%, and a tensile strength of *σ* = 55.5 MPa, all of which surpass values reported for conventional epoxy-based composites. These results confirm the ability of the system to store and retrieve electric energy under direct current (*dc*) fields, while maintaining mechanical robustness and thermal stability due to synergistic interactions between the epoxy matrix and the Fe(II) complex. The multifunctional behavior of the composites underscores their potential as advanced materials for integrated dielectric, piezoelectric, and energy storage and harvesting applications.

## 1. Introduction

The development of next-generation energy systems demands materials that are not only efficient and sustainable but also multifunctional, capable of simultaneously fulfilling multiple roles such as mechanical support, electrical response, energy conversion, and storage. In this context, polymer-based hybrid nanocomposites have gained significant attention due to their inherent structural versatility and capacity to integrate multiple physical phenomena within a single material platform [1,2,3,4,5,6]. Recent comprehensive reviews summarize how polymer-based composites have become a leading platform for integrating electrical energy storage and transduction alongside mechanical performance, and they highlight the major research thrusts, namely high-energy-density dielectrics, piezoelectric nanogenerators, redox-active molecular systems for charge storage, and mechanically robust matrices for device integration [4,6,7]. Among the most promising functionalities sought in such systems are: (i) enhanced dielectric response, which is critical for capacitor and insulation technologies; (ii) piezoelectric activity, enabling conversion of mechanical stress into electrical signals for sensing or energy harvesting; (iii) the ability to store and retrieve electrical energy, relevant to applications in flexible electronics and micro-energy systems; and (iv) robust mechanical stability, necessary for enduring operational stresses and environmental fluctuations in real-world applications such as wearable or structural-integrated electronics [8,9,10].

In recent years, significant research has been directed toward the development of epoxy-based nanocomposites for dielectric and piezoelectric applications. The inclusion of high-permittivity ceramic fillers like barium titanate (BaTiO_3_) and zinc oxide (ZnO) has demonstrated promising results in enhancing the dielectric permittivity and piezoelectric response of these composites [11,12]. While the inclusion of high-permittivity ceramic fillers such as BaTiO_3_ and ZnO can significantly increase *ε′*, numerous reviews emphasize the persistent trade-offs which include filler agglomeration, severe interfacial mismatch, percolation-induced leakage and increased dielectric loss, and difficulty preserving mechanical processability, challenges that motivate alternative molecular or nanoscale design [13,14,15]. In other words, achieving all these functions simultaneously in a lightweight, processable, and environmentally friendly material remains a considerable challenge. Traditional approaches often require the combination of multiple distinct filler phases, such as ceramic nanoparticles for piezoelectricity, conductive or high-permittivity particles for dielectric enhancement, and flexible polymers for mechanical integrity, each introducing complex interface chemistry, dispersion difficulties, and trade-offs between functionality and processability.

Metal-oxide or conductive fillers can raise the permittivity of polymer dielectrics but often at the expense of increased loss and reduced breakdown strength due to interfacial conduction and percolation effects [16,17,18,19]. Coordination compounds are a largely untapped category of functional fillers with significant potential in energy materials. To decouple permittivity enhancement from conduction pathways, to explore discrete, redox-active coordination complexes as molecular fillers is interesting, yet unexplored. Among these, low-spin Fe(II) is attractive because they are diamagnetic d^6^ (minimizing magnetic losses), air-stable solids accessible from commoditized Fe salts under mild conditions, and it exhibits a reversible Fe(III)/Fe(II) couple with tunable kinetics and potentials that can promote interfacial charge storage without a percolation network [20,21]. In this study it is introduced a novel class of fillers based on coordination chemistry, namely tris(2,2′-bipyridine)iron(II) sulfate, [Fe(bpy)_3_]SO_4_, which act as model molecular, diamagnetic, redox-active filler incorporated into a green epoxy resin matrix to test the hypothesis that such complexes can raise dielectric response while maintaining low loss tangent and high energy storage by avoiding electronic percolation and magnetic loss. [Fe(bpy)_3_]^2+^ is a redox-active, low-spin d^6^; complex known for its strong metal-to-ligand charge transfer (MLCT) transitions and tunable electrochemical behavior [22,23], making it a candidate for energy storage and optoelectronic applications. Importantly, recent investigations have highlighted the reversible redox properties and coordination stability of [Fe(bpy)_3_]SO_4_ under ambient conditions, suggesting that this filler could provide both static dielectric enhancement and dynamic charge storage capabilities, which together contribute to energy storage and dielectric behavior. In flow battery systems, for example, [Fe(bpy)_3_]^2+^ has been employed as a catholyte due to its high redox potential and low-cost synthesis from earth-abundant elements. Furthermore, the presence of polar bipyridine ligands can facilitate local dipolar reorientations, potentially contributing to enhanced dielectric relaxation processes within a polymer matrix. Incorporating such a filler into an environmentally friendly epoxy matrix aligns with the principles of green chemistry and material sustainability. The selected epoxy is a green thermosetting epoxy resin with excellent corrosion resistance, low moisture absorption, good thermomechanical properties, high stability, ease of processing, and low cost. The homogenous dispersion of the Fe(II) complex within the matrix, as evidenced by SEM analysis, avoids common issues associated with nanoparticle agglomeration and ensures consistent functional response across the bulk material.

Compared to prior works utilizing fly ash [24], barium titanate, and/or magnetite [25,26] as fillers, the use of a molecular coordination complex represents a conceptual shift toward molecular-level tunability of composite properties. Notably, the broadband dielectric spectroscopy (BDS) data revealed a triple relaxation behavior, indicating multiscale interfacial and dipolar dynamics, which confirms that the Fe(II) complex participates actively in the dielectric response and opens new avenues for tailoring relaxation phenomena in polymer nanocomposites. Additionally, the Fe(II)-based filler contributes to both energy storage and piezoelectric functionality. The observed piezoelectric coefficient (*d*_33_) values compare favorably with previously reported hybrid systems containing BaTiO_3_ or ZnO, despite the absence of a classical piezoelectric ceramic phase. This enhancement is attributed to the dynamic realignment of the polar bipyridine moieties under mechanical stimuli, an effect rarely harnessed in epoxy-based systems.

It is evident that the current work introduces a novel, eco-friendly, sustainable, and multifunctional nanocomposite material system that integrates a redox-active coordination complex into a green epoxy matrix. This approach not only advances the fundamental understanding of dielectric and piezoelectric behavior in such systems but also provides a pathway toward the design of lightweight, cost-effective, and sustainable materials for energy harvesting and storage applications.

## 2. Materials and Methods

### 2.1. Synthesis of [Fe(bpy)_3_]SO_4_

The coordination complex with the molecular formula [Fe(bpy)_3_]SO_4_ (abbreviated as Fe-bpy), where bpy = 2,2′-bipyridine, was synthesized from the reaction of FeSO_4_·7H_2_O and 2,2′-bipyridine, both purchased from Sigma Aldrich (Taufkirchen, Germany), following adapted protocols from the literature [22]. Briefly, a solution of FeSO_4_ (9 g, 32.9 mmol in 50 mL of H_2_O) and 2,2′-bipyridine (15.4 g, 98.7 mmol in 50 mL of acetonitrile) were combined in a 250 mL beaker to give a deep red solution that was stirred for 4 h at room temperature. The solvents were removed under vacuum with rotary evaporation, and the resulting powder was washed three or four times with 20 mL of CH_2_Cl_2_. The red solid was dried under vacuum overnight. Materials (reagent grade) and solvents were used as received, without further purification.

### 2.2. Fabrication of Filaments

For the fabrication of the nanocomposites, the entropy resins consisted of the epoxy prepolymer and curing agent marketed under the brand names “ONE Epoxy resin (High bio-based laminating epoxy)” and “ONE Resin and ONS (SLOW Hardener)”, respectively. The chemical structures of the epoxy resin and hardener are not disclosed by the manufacturer (Entropy Resins, North Walsham, UK), likely for commercial reasons. However, according to the product datasheet, the epoxy prepolymer primarily contains the following ingredients: 4,4′-isopropylidenediphenol, oxirane, and mono[(C12–14-alkyloxy)methyl]. The main ingredients of the hardener, as stated in its datasheet, are polyoxypropylenediamine, trimethylhexamethylenediamine, and 4,4′-methylenebis(cyclohexanamine). According to the information provided by Entropy Resins, more than 70% of the chemicals used are environmentally friendly. Fe-bpy/epoxy nanocomposites with filler contents of 1, 3, 5, 7, and 10 phr (mass parts of filler/100 mass parts of resin, %w/100 w)) were fabricated. A reference specimen of neat resin was also prepared. The fabrication began by blending pre-measured quantities of Fe-bpy particles into the resin prepolymer at room temperature. The mixture was then sonicated (Elma S30H, Elmasonic, sweep mode, 280 W) for 10 min at 50 °C to ensure uniform particle dispersion and, although unlikely in this case, to minimize agglomeration that could impair composite properties. After sonication, the mixture was cooled to room temperature, followed by the addition of a curing agent at a 2:1 (*w*/*w*) ratio of epoxy prepolymer to curing agent. This blend was again sonicated for 10 min at room temperature before being poured into silicone molds and allowed to cure for seven days at ambient conditions. Finally, the specimens underwent post-curing at 100 °C for 4 h. Specimen sets were prepared in geometries appropriate for each experimental technique used. For comparison, an epoxy specimen without filler was also fabricated. The dispersion quality of the particles within the epoxy matrix and the overall morphology of the fabricated specimens were characterized using scanning electron microscopy (SEM) on a Carl Zeiss EVO MA 10 instrument (Zeiss, Oberkochen, Germany).

Dynamic mechanical behavior was evaluated using a TA Instruments Q800 dynamic mechanical analyzer (DMA) (TA Instruments, Lukens Drive, New Castle, DE, USA) in three-point bending mode. Tests were conducted over a temperature range from ambient to 100 °C, at a heating rate of 5 °C min^−1^, with an oscillation frequency of 1 Hz.

The static mechanical properties of the fabricated nanocomposites were evaluated using an Instron 5582 tester (Instron, Norwood, MA, USA) at room temperature with a tensile rate of 5 mm/min. Their electrical behavior, including dielectric response and AC conductivity, was analyzed through Broadband Dielectric Spectroscopy (BDS) using an Alpha-N Frequency Response Analyzer, Phecos System, BDS 1200 dielectric cell (Novcontrol Technologies, GmbH & Co. KG, Aubachstr. Montabaur, Germany), and Windeta software 3.0, all supplied by Novocontrol Technologies. Measurements were carried out under isothermal conditions between 30 °C and 160 °C, with the applied field frequency ranging from 10^−1^; Hz to 10^6^ Hz at Vrms = 1 V, increasing the temperature in 5 °C steps after each frequency scan. AC dielectric tests followed ASTM D150 standards [27]. The stability/behaviour of all samples under humidity was also investigated. In particular, following the established protocol ASTM D570 [28], the samples were immersed in distilled water for 24 h and further analyzed. The samples were then dried again and reanalyzed to test if the behaviour is similar or not to the behaviour of the pristine samples. The procedure steps briefly include drying of the samples at 60 °C for 24 h and subsequent immersion of the samples in triple-distilled water for 24 h at 23 °C. The samples were then dried with a tissue, and the dielectric response was recorded. Before and after immersion, the mass of the samples was determined. The water absorption uptake of the samples was determined by the: (%): [(*m_after immersion_* − *m_initial_*)/*m_initial_*] × 100. After the measurement of the “wet” samples, subsequent drying at 60 °C for 24 h was followed; the final mass of the samples was also measured again after drying, which was approximately the same as the pristine samples.

Energy storage and release capabilities were examined through DC measurements by recording time-dependent charging and discharging currents with a High-Resistance Meter DC (Agilent 4339B, Analytical Instruments SA, Chalandri, Greece). Tests were performed at room temperature under two voltage levels (100 and 200 V), with samples placed between the electrodes of a parallel-plate capacitor. Each charging cycle lasted 60 s, the charging procedure was followed by a 300 s discharging period; a short-circuit discharge step was also applied beforehand to eliminate any residual charges. DC tests followed ASTM D257 specifications [29]. Further details of the setup and procedures are provided in [26,30]. Piezoelectric characterization was carried out using a PM300 piezometer (Piezo Test LTD, London, UK), with the longitudinal piezoelectric coefficient (*d*_33_) determined under a 0.25 N applied force at a frequency of 110 Hz.

All experiments were performed in triplicate. In each measurement a deviation of <0.2% for each value was recorded, and therefore the mean values are shown, while error bars are shown where needed.

## 3. Results and Discussion 

**Figure 1:**
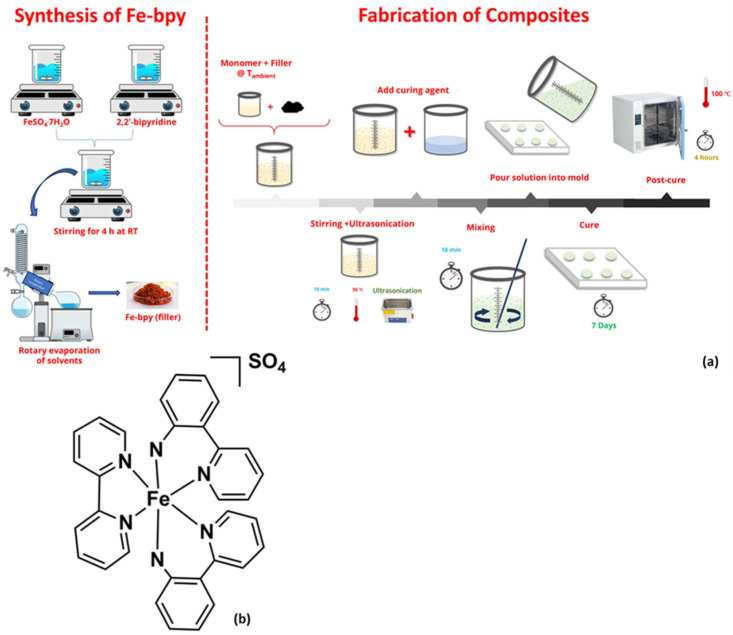
(**a**) Synthesis of the coordination complex [Fe(bpy)_3_]SO_4_ and fabrication of the composites with the green resin. (**b**) The molecular structure of[Fe(bpy)_3_]SO_4_.

The selection of the [Fe(bpy)_3_]SO_4_ (tris(2,2′-bipyridine)iron(II) sulfate) coordination complex as the functional filler in this study was driven by its unique physicochemical properties that align closely with the multifunctional requirements of next-generation energy materials. As a redox-active complex with a well-characterized metal-to-ligand charge-transfer (MLCT) band and a polar molecular structure, [Fe(bpy)_3_]^2+^ offers a compelling combination of electronic tunability, dielectric responsiveness, and potential for energy storage and piezoelectric-like behavior. The three bidentate bipyridine ligands surrounding the Fe(II) center not only provide molecular rigidity and stability but also introduce significant dipolar character that can interact favorably with the applied electric field and the polymer matrix environment. To translate these molecular-level features into macroscopic composite performance, the complex was synthesized via a straightforward coordination reaction between FeSO_4_ and 2,2′-bipyridine in aqueous solution, followed by precipitation and purification steps. The resulting deep red powder was then incorporated into a bio-based epoxy resin and cured, ensuring homogeneous dispersion and preservation of the Fe(II) oxidation state. This synthetic strategy represents a conceptually novel approach: rather than relying on conventional ceramic or oxide fillers, we demonstrate that a well-designed coordination complex can be molecularly engineered and embedded within a polymer matrix (Figure 1) to impart multiple functionalities, dielectric enhancement, energy storage capacity, and mechanical reinforcement, through controlled interfacial interactions and dynamic dipolar activity.

**Figure 2:**
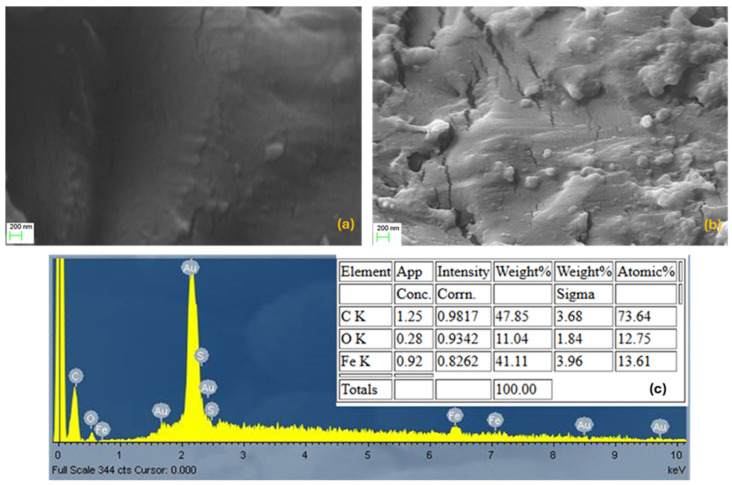
SEM images of the (**a**) 3 phr Fe-bpy/epoxy and (**b**) 10 phr Fe-bpy/epoxy composites. (**c**) EDX analysis of the 10 phr Fe-bpy/epoxy composite.

The SEM images in Figure 2a,b illustrate the morphology of the fabricated composites at 3 and 10 phr. The results indicate that Fe-bpy microparticles are well-dispersed within the matrix, with no significant clustering observed. Even at the highest filler loading of 10 phr, only a small number of agglomerates become apparent. The existence of the Fe-bpy coordination complex is also evident in EDX analysis. The EDX analysis in different parts of the sample revealed a similar percentage of Fe (i.e., percentage of the coordination complex), and a representative EDX spectrum and results are presented (Figure 2c).

### 3.1. Dielectric Properties

Owing to their intrinsically low free-charge carrier density, polymer composites exhibit electrical insulating behavior, with their dielectric properties predominantly governed by relaxation phenomena under alternating current (AC) conditions. These relaxations arise from the reorientation dynamics of permanent and induced dipoles, which are intrinsically linked to space-charge migration and the presence of polar functionalities along the polymer backbone.

**Figure 3:**
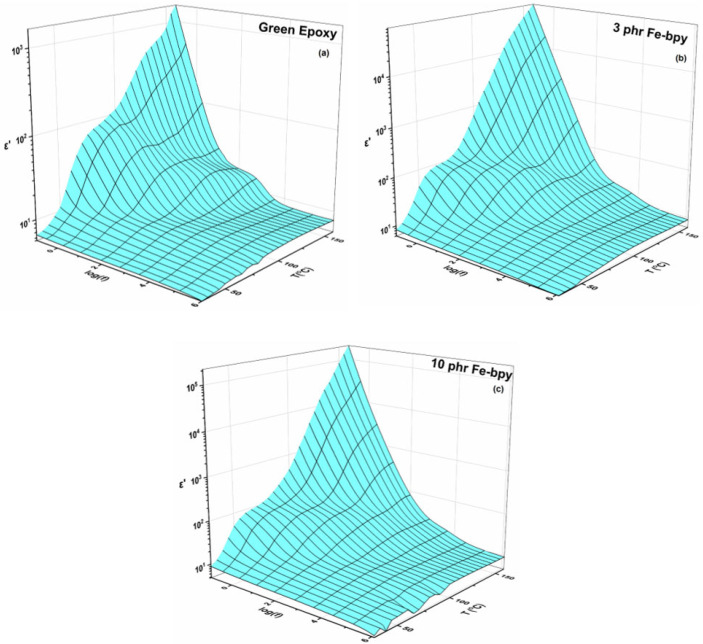
Real part of dielectric permittivity (*ε′*) as a function of frequency and temperature for the green epoxy resin (**a**) and the composites with (**b**) 3 phr and (**c**) 10 phr of Fe-bpy content.

**Figure 4:**
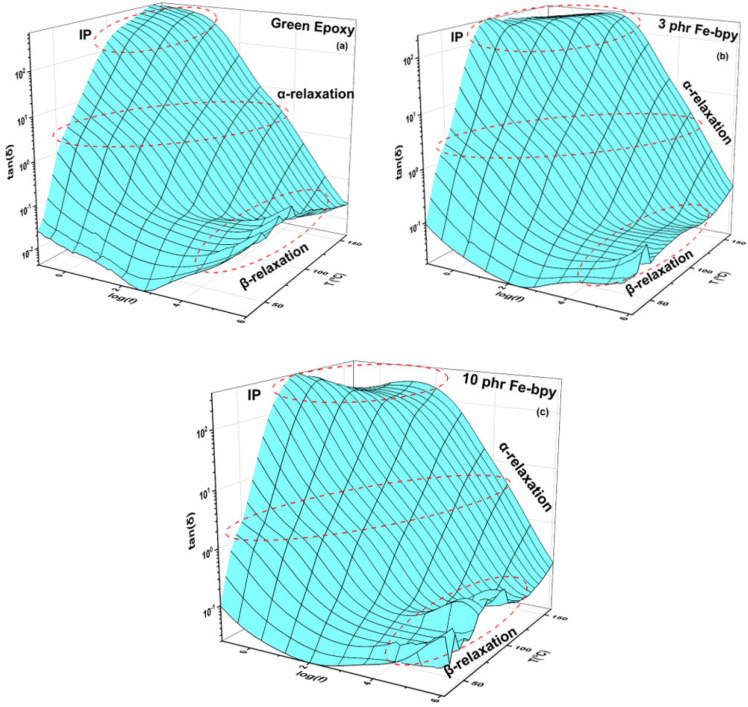
Loss tangent (tan*δ*) as a function of frequency and temperature for the green epoxy resin (**a**) and the composites with (**b**) 3 phr and (**c**) 10 phr of Fe-bpy content.

The dielectric response of nanocomposite systems incorporating 3 phr and 10 phr Fe-bpy as the reinforcing phase compared to the neat epoxy resin is presented in Figure 3 and Figure 4 as three-dimensional plots of the real part of the dielectric permittivity (*ε′*) and the loss tangent (tan δ) as functions of temperature and frequency. As shown in Figure 3b,c, *ε′* decreases systematically with increasing frequency, reflecting the inability of dipoles, both permanent and induced, to follow rapid alternations of the applied field due to their inherent inertia. In contrast, at low frequencies, dipoles have sufficient time to align with the field, yielding elevated *ε′* values. Thermal activation further enhances dipole mobility, resulting in the highest *ε′* values at low frequencies and elevated temperatures. The 3D spectra also reveal three distinct step-like transitions, indicative of multiple relaxation processes.

Loss tangent spectra (Figure 4) provide enhanced resolution of these processes, revealing three well-defined peaks. The first is attributed to interfacial polarization (IP) at the epoxy/Fe-bpy interface, where the accumulation of unbound charges gives rise to large interfacial dipoles. The effect intensifies at low frequencies and high temperatures; the substantial size of these dipoles imparts considerable inertia, necessitating longer alignment times and greater thermal energy, thereby producing elevated permittivity in this regime. The second process corresponds to *α*-relaxation, associated with the glass-to-rubber transition of the epoxy matrix. Here, the cooperative motion of cross-linked macromolecular segments enables large portions of the polymer chains to reorient under the applied field. The third, *β*-relaxation, is detected in the high-frequency domain and arises from localized reorientation of small polar side groups, constituting a secondary relaxation with comparatively low intensity.

**Figure 5:**
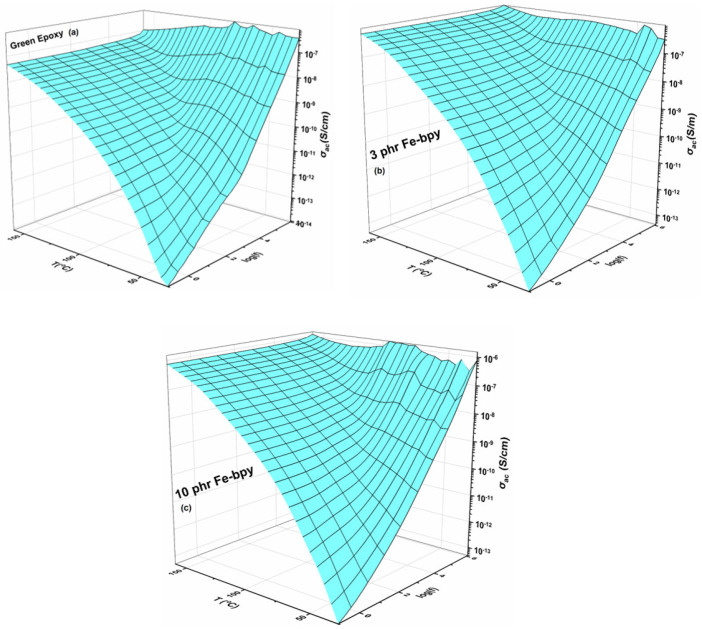
AC conductivity as a function of frequency and temperature for the green epoxy resin (**a**) and the composites with (**b**) 3 phr and (**c**) 10 phr Fe-bpy content.

AC conductivity analysis of the same composites and the green resin was also performed (Figure 5). The conductivity exhibits strong frequency and temperature dependence, with the influence of temperature being particularly pronounced at low frequencies, indicating a thermally activated conduction mechanism. The insulating polymer matrix, combined with semiconducting inclusions, yields low baseline conductivity that rises with temperature. At low frequencies, the slowly varying field allows charge carriers to traverse greater distances; however, high potential barriers within the insulating matrix limit mobility. Only a small fraction of carriers participate in this regime, but thermal activation enables some to surmount these barriers, increasing conductivity [31].

At high frequencies, the AC conductivity increases exponentially in a temperature-independent manner, consistent with a hopping conduction mechanism in which carriers (electrons, ions, or polarons) move between adjacent sites separated by low potential barriers [31]. In this regime, the number of active carriers increases sharply, although the distance each can travel is significantly reduced. The frequency dependence of AC conductivity at a constant temperature in these disordered systems follows the universal law [32,33]:

(1) $$\sigma_{AC}\left(\omega \right)=\sigma_{DC}+{A(\omega )}^{s}$$

where *σ_DC_* is the DC limiting value, *ω* is the field’s angular frequency, and *A* and *s* are temperature- and filler-dependent parameters.

Increasing temperature shifts the exponential portion of the AC conductivity curves upward across the frequency range. In addition, shoulder-like features in the intermediate frequency region can be attributed to the relaxation mechanisms described above, reflecting the interplay between interfacial polarization, *α*-relaxation, and *β*-relaxation in governing the charge transport behavior.

**Figure 6:**
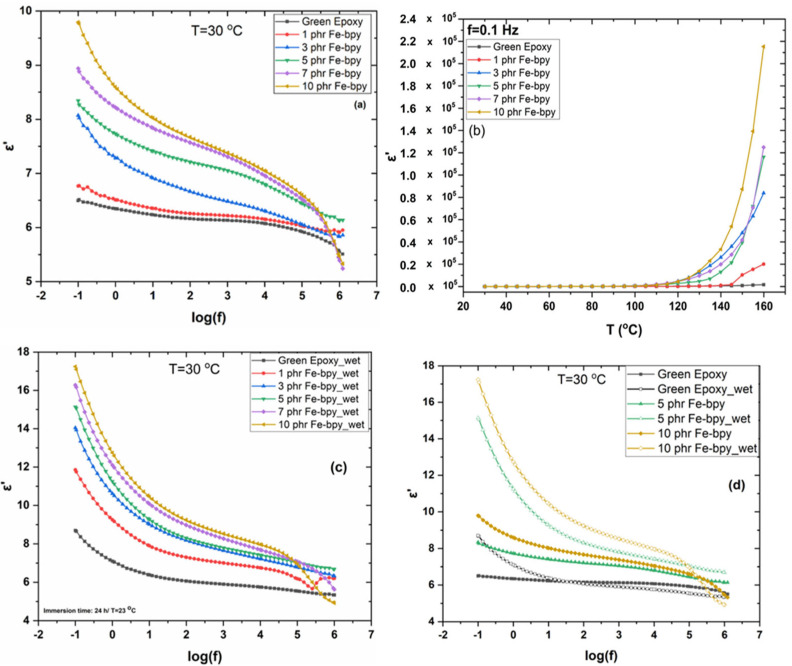
(**a**) Real part of dielectric permittivity (*ε′*), at 30 °C, as a function of frequency, and (**b**) real part of dielectric permittivity (*ε′*), at 0.1 Hz, as a function of temperature. (**c**) Real part of dielectric permittivity (*ε′*), at 30 °C, as a function of frequency for the samples after immersion in water, and (**d**) comparison of the samples of the green resin and 5 and 10 phr Fe-bpy/resin before and after immersion.

Dielectric permittivity (*ε′*) is a fundamental material property that quantifies how much electric energy a material can store when subjected to an external electric field. In simple terms, it measures how polarizable a material is. The real part of permittivity (*ε′*) represents the stored energy (how much electric flux is supported per unit field), while the imaginary part (*ε″*) corresponds to dielectric losses (energy dissipated as heat). Figure 6a presents the frequency dependence of the real part of the dielectric permittivity (*ε′*) at 30 °C for all examined systems. An increase in *ε′* with Fe-bpy loading is evident, reflecting an additional reinforcing effect of the filler. Across the entire frequency range, all reinforced composites exhibit higher *ε′* values than the neat epoxy. The decrease in *ε′* with increasing frequency is consistent with a reduced ability of dipoles to align with the rapidly alternating electric field, leading to diminished polarization at high frequencies. Indicatively, the *ε′* at the low-frequency limit increased gradually as a function of the Fe-bpy content, reaching the value from 6.5 (neat epoxy) to 9.8 (10 phr), while at 1 kHz this value increases from 6.1 (neat epoxy) to 7.4 (10 phr).

The temperature dependence of *ε′* at a fixed frequency of 0.1 Hz is shown in Figure 6b. Above ~120 °C, a pronounced increase in *ε′* is observed, attributed to interfacial polarization (IP), which is promoted at elevated temperatures and similar to all specimens, revealing the homogeneity of the samples [34,35]. Increased Fe-bpy content amplifies IP, resulting in higher *ε′* values across both frequency and temperature domains. Given that Fe-bpy possesses a higher dielectric permittivity than the polymer matrix, reinforced systems maintain superior *ε′* throughout the measured spectra.

The stability/behaviour of all samples under humidity was also investigated. In particular, following the established protocol, ASTM D570 [28], the samples were immersed in distilled water for 24 h and further analyzed. The samples were then dried again and reanalyzed to test if the behaviour is similar or not to the behaviour of the pristine samples. The water absorption uptake in the samples is less than 0.5%, and a gradual increase in the uptake is noticed as the loading of Fe-bpy is increased (Table 1), which is rationalized by taking into account the hydrophilic organic bipyridine ligands coordinated to the Fe metal ion in the coordination complex. It is evident that the dielectric response of the Fe-bpy/resin composites of all samples (green resin, 1–10 phr Fe-bpy/resin composites) is different after the immersion. In particular, the *ε′* under the same experimental conditions of the measurement in the pristine samples is higher in all cases compared to the initial samples (Figure 6c). This is attributed to the fact that the co-presence of the water molecules within the samples increases the dielectric response of the samples. The difference of the *ε′* between the pristine samples and the “wet” samples (*ε’_rel_*= [(*ε’_wet_* − *ε’_dry_*)/*ε’_dry_*] × 100) was calculated (Table 1). For instance, the *ε′* of the pristine and the wet samples at 5 and 10 phr at the lowest frequency is increased from 8.3 to 15.1 and from 9.8 to 17.3, respectively. After the samples were dried, the absorbed water was removed, and the dielectric response was similar to the pristine samples (Figure 6d).

Dielectric responses can be analyzed through several formalisms, including dielectric permittivity, AC conductivity, and electric modulus. Electric modulus eliminates the parasitic effect of electrode polarization and all relevant capacitances and is defined as the inverse quantity of complex permittivity:

(2) $$M^{*}=\frac{1}{\varepsilon^{*}}=\frac{1}{\varepsilon^{\prime }-j\varepsilon^{\prime \prime }}=\frac{\varepsilon^{\prime }}{\varepsilon^{\prime 2}+\varepsilon^{\prime \prime 2}}+j\frac{\varepsilon^{\prime \prime }}{\varepsilon^{\prime 2}+\varepsilon^{\prime \prime 2}}=M^{\prime }+jM^{\prime \prime }$$

where *ε’* and *Μ’*, and *ε”* and *M″* are the real and imaginary parts of the dielectric permittivity, and the electric modulus, respectively [35].

**Figure 7:**
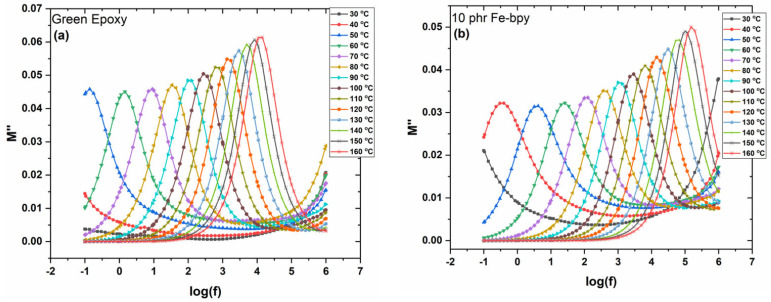
Electric modulus loss index versus frequency for (**a**) neat epoxy and (**b**) the composite with 10 phr of Fe-bpy content, at various temperatures.

Figure 7a,b display the loss modulus (*M″*) spectra for the neat epoxy and the composite containing 10 phr Fe-bpy, measured at frequencies corresponding to the transitions identified in the *ε′* 3D spectra, over various temperatures. The dominant peaks correspond to the glass-to-rubber *α*-relaxation. As temperature increases, these peaks shift toward higher frequencies in accordance with the frequency–temperature superposition principle. Notably, a secondary peak emerges at the high-frequency edge, corresponding to *β*-relaxation, which arises from localized motions of small polar side groups.

**Figure 8:**
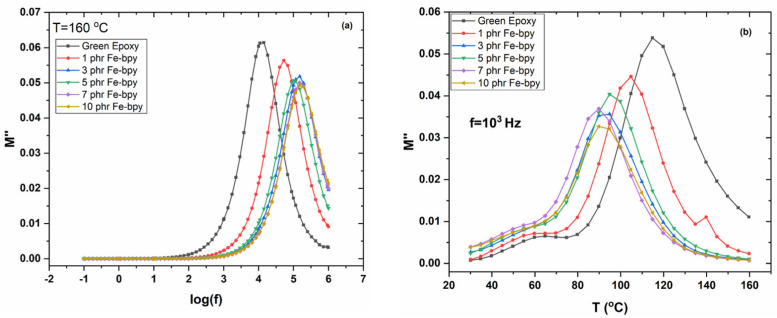
(**a**) Electric modulus loss index versus frequency, at 160 °C, and (**b**) electric modulus loss index versus temperature, at 10^3^ Hz, for all examined systems.

The frequency dependence of the imaginary part of the electric modulus (*M″*) at 160 °C for all systems is shown in Figure 8a. The presence of a distinct loss peak in all cases confirms the occurrence of *α*-relaxation, associated with the glass transition temperature (T_g_) of the amorphous polymer phase. Near T_g_, absorbed thermal energy enables cooperative segmental motions of substantial portions of the polymer chains. Variations in peak position at constant temperature reflect differences in polymer–filler interactions: a shift toward lower frequencies indicates weaker interactions, while a shift to higher frequencies suggests stronger interactions [36,37,38,39].

Figure 8b plots *M″* versus temperature at 1 kHz. All spectra exhibit two distinct peaks: the main peak, assigned to *α*-relaxation, shifts toward higher temperatures with increasing Fe-bpy content, implying an elevation in T_g_ resulting from strong interfacial adhesion between the filler and the polymer matrix. The secondary, weaker peak at lower temperatures corresponds to *β*-relaxation, attributed to the rearrangement of polar side groups along the polymer backbone.

### 3.2. Energy Storage and Harvesting Properties

**Figure 9:**
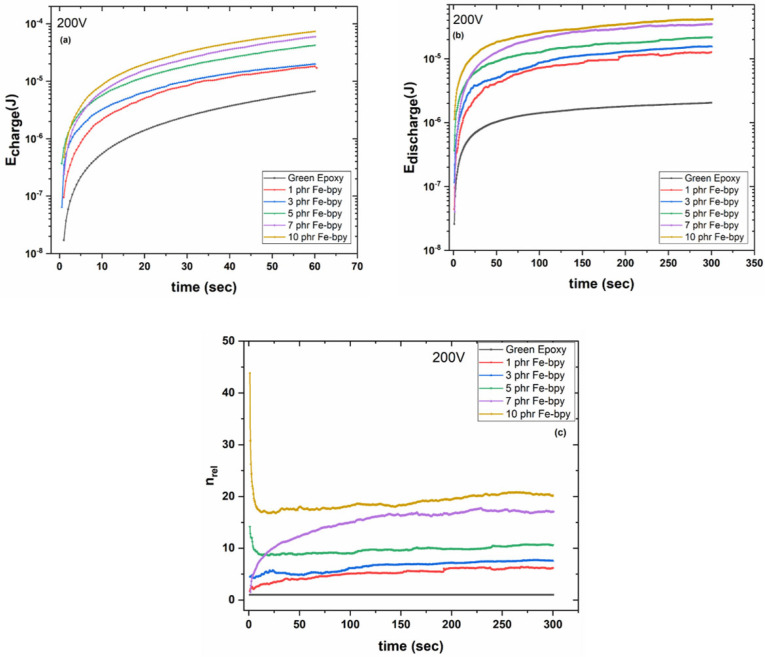
Variation in (**a**) storing energy (*E_charge_*), (**b**) retrieving energy (*E_discharge_*), and (**c**) the relative coefficient of energy efficiency (*n_rel_*), as a function of time, for all studied systems, at charging voltage of 200 V.

Figure 9a,b illustrate the time-dependent behavior of stored and retrieved energy for all investigated composites under an applied charging voltage of 200 V. The incorporation of Fe-bpy particles, which behave as a dispersed network of microscopic capacitive elements, leads to a notable increase in both energy storage and retrieval capabilities with increasing filler content. This interconnected filler network effectively constitutes a microscale active system capable of rapid charge–discharge cycles. The stored and retrieved energy values were calculated by integrating the time-dependent charge/discharge current functions via Equation (3):

(3) $$E=\frac{1}{2}\frac{Q^{2}}{C}=\frac{1}{2}\frac{{\left[\int I\left(t\right)dt\right]}^{2}}{C}$$

where *E* is the stored/retrieved energy at the composite, *Q* the amount of charge, *I(t)* the charging or discharging current and *C* is the capacitance of the composite as derived by the BDS measurements at low frequency [7,13].

Given the predominantly insulating nature of the matrix, charge carriers injected under an applied voltage/electric field are generally unable to traverse the entire specimen. However, at elevated temperatures, carriers may acquire sufficient thermal energy to overcome potential barriers, thereby enhancing electrical conductivity. At room temperature, only a limited fraction of these carriers possesses the necessary energy to surmount the barriers, resulting in constrained charge mobility and correspondingly low conductivity.

An increase in the magnitude of the applied voltage/electric field during the charging process may reduce the height of these potential barriers, facilitating greater carrier mobility. Under such conditions, carriers follow a trapping/detrapping mechanism as they navigate the extensive interfacial regions between the matrix and filler. This process, while enhancing conduction, also leads to elevated leakage currents, which in turn diminish the proportion of energy that can be effectively recovered [26,30,39].

To quantify the effectiveness of energy storage and retrieval, the energy efficiency coefficient (*n_eff_*) is introduced. This parameter, defined by Equation (4), offers a means to evaluate the relative efficiency of the charge–discharge process across the different composite systems.

(4) $$n_{rel}=\frac{E_{retr,comp}}{E_{retr,matrix}}$$

where *E_retr,comp_* and *E_retr,matrix_* are the retrieved energies from a composite and the matrix under the same charging voltage and at the same instance, respectively. Figure 9c illustrates the time dependence of the relative coefficient of energy efficiency for all systems examined in this study at 200 V. It is evident that the ability to retrieve energy is analogous to the filler content being increased up to 44 times compared to the ability of the neat green resin to restore energy. For instance, at *t* = 0 s, the zero value of energy efficiency coefficient (*n_rel_*) rose to 44 at 10 phr Fe-bpy composite, while even after *t* = 300 s, the energy efficiency coefficient (*n_eff_*) value remained as high as 19.5.

In previous work, epoxy nanocomposites incorporating ceramic fillers such as ZnTiO_3_ and BaTiO_3_ demonstrated energy storage and retrieval enhancements in the range of 30–50× compared with the neat resin, depending on filler type and loading [40]. These findings align closely with the present system. As Figure 9a,b demonstrate, the inclusion of Fe-bpy particles behaves as a network of microscopic capacitive elements, enhancing both energy storage and retrieval in proportion to filler content, culminating in energy retrieval improvements of up to 44× relative to the neat green resin (Figure 9c). Beyond achieving high permittivity, our particles also contribute redox-mediated trapping/detrapping pathways, offering an efficient, rapid-charge architecture that leverages local conduction, thus enhancing functionality while maintaining insulation.

### 3.3. Piezoelectricity Properties

**Figure 10:**
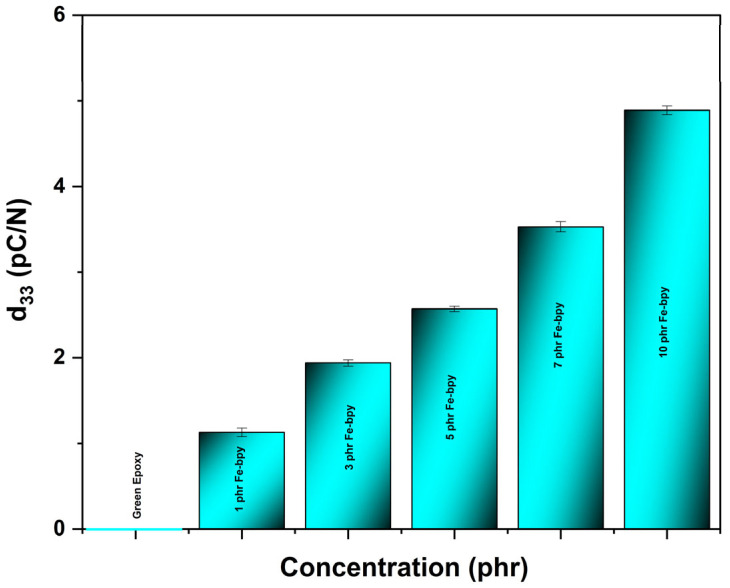
Piezoelectric coefficient (*d*_33,_ pC/N) as a function of Fe-bpy content.

In terms of piezoelectricity, the longitudinal piezoelectric coefficient (*d*_33_) was determined for all investigated specimens. As shown in Figure 10, *d*_33_ (pC/N) increases with the Fe-bpy content reaching the maximum value of 5.1 pC/N in 10 phr Fe-bpy, and they are significantly higher compared to the zero value of the green resin. This increased piezoelectric response is noticeable also compared to other composites having other fillers and the same resin. The increase in *d*_33_; correlates with the enhanced permittivity of the composite, indicating that the interfacial polarization mechanisms responsible for capacitance enhancement also contribute to the observed piezoelectric response. The neat epoxy is non-piezoelectric. Incorporation of Fe-bpy produces a clear, monotonic piezoelectric response: *d*_33_ = 1.1 pC/N (1 phr), 1.9 pC/N (3 phr), 2.6 pC/N (5 phr), 3.5 pC/N (7 phr), and 5.1 pC/N (10 phr). Therefore, at a 10 phr sample, *ε′* increased by 2 orders of magnitude compared to the neat polymer, accompanied by a rise in *d*_33_ from zero to 5.1 pC/N, suggesting a proportional contribution of interfacial dipole reorientation under stress. These magnitudes are comparable to or better than polymer/ceramic nanoparticle composites at similar filler loadings (typical polymer–ceramic composites report *d*_33_ ≈ 2–4 pC/N under similar conditions). The origin is the polar Fe-bpy complex plus interfacial polarization producing dipolar alignment under applied stress. The monotonic increase of the composite d_33_; with filler content is therefore attributable to the combined contributions of the intrinsic piezoelectricity of the Fe–bpy complex and the enhanced interfacial polarization within the polymer matrix. Beyond this dielectric effect, the Fe(bpy)_3_^2+^ complex introduces reversible Fe(II)/Fe(III) redox activity, which facilitates dynamic charge redistribution and stabilizes mechanically induced dipoles. This dual contribution, dielectric polarization and molecular redox transitions, differs fundamentally from conventional ceramic-filled polymer nanocomposites, where *d*_33_ enhancements are primarily attributed to interfacial polarization of high-permittivity inclusions [41]. The present system, therefore, exemplifies a new design principle in piezoelectric composites, aligning with recent calls to move beyond classical filler paradigms toward molecularly engineered dielectric systems [42,43,44]. In previous investigations, epoxy-based composites filled with conventional piezoelectric ceramics typically exhibit modest *d*_33_ enhancements [45,46]. For example, composites containing BaTiO_3_ fillers often reach *d*_33_ in the range of 1–3 pC/N at moderate loadings, and approach ~ 5 pC/N only at very high filler volume fractions (>50 vol%) [47]. In contrast, in this case, a progressive increase was noticed in *d*_33_ with increasing Fe-bpy content, attaining ~ 5.1 pC/N at 10 phr, while the neat resin exhibits zero piezoelectric response (Figure 10). The comparative magnitude and filler-loading relationship place our results on par with, or exceeding, those earlier epoxy/ceramic composites 111, especially considering our much lower filler percentages. More importantly, the Fe-bpy system combines capacitive and redox-mediated charge storage mechanisms, offering rapid charge/discharge cycling unlike the inert ceramic fillers used previously.

### 3.4. Thermomechanical Properties

**Figure 11:**
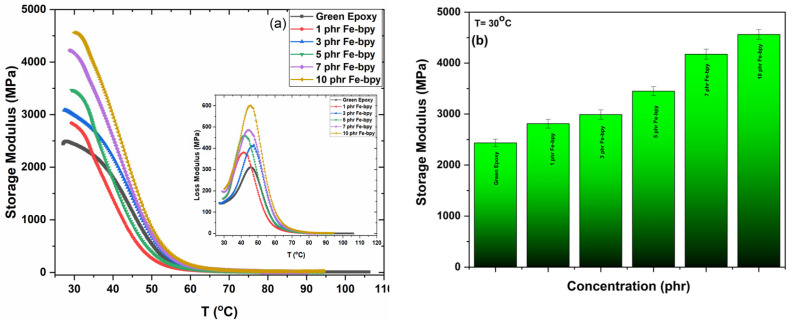
(**a**) Storage modulus and loss modulus (inset) as a function of temperature for all studied systems. (**b**) Max storage modulus E’ versus filler content for all studied systems at 30 °C.

The thermomechanical behavior of all studied systems, as determined by dynamic mechanical analysis (DMA), is shown in Figure 11. The storage modulus as a function of temperature for varying Fe-bpy nanoparticle contents is presented in Figure 11a. In all cases, the storage modulus decreases markedly with increasing temperature, reflecting the transition from the rigid, glassy state to a more compliant, rubbery-like regime. The systematic increase in storage modulus at the room temperature with higher nanoparticle loading is more apparent in Figure 11b, which highlights the reinforcing capability of Fe-bpy and the fine particle dispersion and strong interfacial adhesion between filler and matrix [12,48]. From the corresponding loss modulus spectra (inset of Figure 11a), it is observed that within the glass transition region, pronounced peaks are observed, representing the dissipation of mechanical energy as heat. These peaks further confirm the viscoelastic relaxation processes associated with the glass-to-rubber transition.

Notably, maintaining high mechanical strength alongside enhanced electrical and piezoelectric performance is crucial for the practical deployment of these materials, ensuring structural integrity and durability under operational stresses while preserving their multifunctional properties.

**Figure 12:**
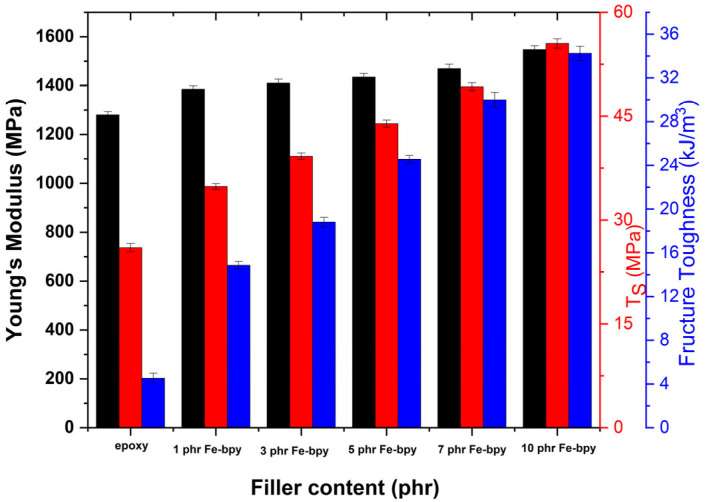
Young’s modulus, tensile strength, and fracture toughness as a function of Fe-bpy content.

Figure 12 shows the results of the static mechanical tests, and specifically Young’s modulus, tensile strength, and fracture toughness, which were determined from the tensile stress–strain plots. It is noticed that the modulus of elasticity increases consistently with increasing Fe-bpy content.

The effect of filler content on tensile strength and fracture toughness is significant, enhancing the reinforcing capability of Fe-bpy in improving mechanical properties. At higher filler contents, both tensile strength and fracture toughness increase several-fold compared to the unfilled polymer matrix. Young’s modulus increases with Fe-bpy loading, with the trend 1280 MPa (neat resin), 1385 MPa (1 phr), 1410 MPa (3 phr), 1435 MPa (5 phr), 1470 MPa (7 phr), and 1548 MPa (10 phr). Overall, Fe-bpy improves the mechanical durability of the nanocomposites, and the incorporated particles do not act as stress concentration points or stress raisers within the epoxy matrix, factors that would otherwise lead to a considerable reduction in both tensile strength and fracture toughness with increasing filler content. This observation is consistent with the results of the DMA testing.

### 3.5. Comparative Analysis of the Dielectric Properties and Energy Storage Performance

Recent progress in polymer-based dielectric nanocomposites emphasizes the trade-off between permittivity, dielectric loss, and efficiency (Table 2) [49]. For example, BaTiO_3_/PVDF-based composites can reach *ε′* ≈ 60–90 at 1 kHz, but typically exhibit tan δ ≈ 0.05–0.10 and energy storage efficiencies of about ~70–75% [50,51]. By contrast, low-loading two-dimensional nanosheet fillers such as Ti_0.87_O_2_ or TiO_2_ in PVDF deliver more moderate permittivity values (*ε′* ≈ 10–12), yet maintain low dielectric losses (tan δ ≤ 0.03) and efficiencies of 60–63% [52,53]. ZrO_2_ nanosheet/PVDF composites follow a similar trend, with *ε′* ≈ 10, tan δ < 0.04, and *η* ≈ 67% [54,55]. Graphene-based systems demonstrate tunable interfacial polarization and band alignment, achieving either moderate *ε′* (≈ 20) with relatively large losses (tan δ ≈ 0.05), and high *η* ≈ 60–80%) [56]. Against this backdrop, our Fe–bpy/epoxy nanocomposites achieve multifunctionality at low filler levels. At 10 phr, *ε′* is ~7.4 (1 kHz, 30 °C), tan δ remains ≤ 0.05, and the energy storage efficiency *η* is ~44% under dc charge–discharge testing (200 V). Although the efficiency is lower than the best-performing nanosheet/PVDF systems, our composites uniquely combine stable dielectric behavior with a measurable piezoelectric coefficient (*d*_33_ = 5.1 pC/N) and preserved mechanical strength (~55 MPa) with a considerable dielectric response compared to literature. This multifunctional performance in a sustainable epoxy matrix highlights an alternative design strategy where electromechanical activity and structural robustness are prioritized alongside moderate dielectric efficiency.

## 4. Conclusions

A redox-active coordination complex, [Fe(bpy)_3_]SO_4_, was successfully integrated into a green epoxy resin to develop multifunctional nanocomposites combining enhanced dielectric, energy storage, piezoelectric, and mechanical properties. SEM confirmed uniform dispersion with minimal agglomeration up to 10 phr filler content. Dielectric measurements showed a systematic increase in *ε′* with loading, reaching maximum values above ~120 °C due to interfacial polarization, and revealed three distinct relaxation processes (IP, *α*, and *β*) from broadband dielectric spectroscopy. AC conductivity indicated thermally activated transport at low frequencies and hopping conduction at high frequencies. Under DC charging at 200 V, stored and retrieved energy increased proportionally with Fe-bpy content, achieving relative energy efficiency up to 44× higher than the neat resin, comparable to high-k ceramic systems but at lower loadings, due to a distributed capacitive network and redox-mediated trapping/detrapping effects. The longitudinal piezoelectric coefficient (*d*_33_) rose from zero in the neat resin to 5.1 pC/N at 10 phr, rivaling values of epoxy/ceramic composites without the need for a classical piezoelectric phase. DMA confirmed improved stiffness in the glassy region and strong filler–matrix adhesion, maintaining mechanical integrity alongside electrical functionality. Overall, the results highlight the potential of molecularly engineered fillers to deliver lightweight, sustainable, and mechanically robust materials for integrated energy harvesting, storage, and sensing applications.

## Figures and Tables

**Table 1:** The water absorption uptake and the relative *ε′*_rel_ before and after the immersion.

Sample	m_after immersion_ − m_initial_ (g)	Absorption (%)	m_final_ (g) After Re-Drying	*ε′*_rel_ Τ = 30 °C, f = 10^−1^ Hz
Neat epoxy	0.0089	0.460	1.9310	0.339
1 phr Fe-bpy	0.0081	0.398	2.0332	0.753
3 phr Fe-bpy	0.0095	0.484	1.9608	0.740
5 phr Fe-bpy	0.0106	0.554	1.9113	0.815
7 phr Fe-bpy	0.0107	0.559	1.9120	0.738
10 phr Fe-bpy	0.0109	0.599	1.8166	0.760

**Table 2:** Evaluation of energy storage properties.

Filler	Polymer	Content	*ε* *’*	tan*δ*	*η* (%)	Ref.
**[Fe(bpy)_3_]SO_4_**	**Green Epoxy Resin**	**3–10 wt.%**	**40.7 (0.1 Hz)/7.8 (1 kHz, 30 °C)**	**0.05**	**44**	**This Study**
BaTiO_3_	P(VDF-TrFE-CTFE)	15%	90.2	0.1	74.2	[51]
BaTiO_3_	PVDF	0.3%	11.9	<0.04	55	[50]
SrTiO_3_	PVDF	1 wt.%	10.66	-	57.2	[57]
NaNbO_3_	P(VDF-HFP)	3%	12	<0.05	70.1	[58]
K_0.5_Na_0.5_NbO_3_	PVDF	3%	12	<0.05	80.2	[59]
Na_0.5_Bi_4.5_Ti_4_O_15_	PVDF	1 wt.%	16	0.1	52.3	[60]
Ca_2_Nb_3_O_10_	PVDF	2.1 wt.%	10.5	-	61.2	[61]
Ca_2_Nb_3_O_10_	P(VDF-HFP)	0.1%	-	-	-	[62]
Sr_2_Nb_2_O_7_	PVDF	5 wt.%	11	<0.05	71	[63]
ZrO_2_	PVDF	1 wt.%	10	<0.04	67.4	[54]
Ti_0.87_O_2_	PVDF	1 wt.%	12	<0.03	60	[52]
TiO_2β_	PMMA/P(VDFHFP)	5 wt.%	10	~0.04	63	[53]
MMT	PVDF	0.2 wt.%	28	0.032	>60	[64]
Na^+^/MMT	PVDF	-	15	<0.02	81	[65]
MoS_2_	PVDF	0.4%	11.3	0.07	~72	[55]
Bi_2_Te_3_	PVDF	10 vol.%	140	0.05	-	[66]
MoS_2_	Chitin	5 wt.%	~9.8	~0.025	>80	[67]
MoS_2_	g-PMMA/PI	3 wt.%	4.2	0.015	61.7	[68]
MoS_2_	P(VDF-CTFE-DB)	2 wt.%	12.9	0.047	83	[69]
Graphene	P(VDF-TrFE-CFE)	0.1 wt.%	~15	~0.04	78.1	[56]
Graphene	P(VDF-CTFE)	0.8 vol.%	24.8	0.06	62	[55]
GO	P(VDF-HFP)	2 wt.%	~11	~0.1	77	[55]
BNNS	P(VDF-TrFE-CFE)	12 wt.%	38	0.03	78	[70]

## Data Availability

Data are available upon request.
